# GEROSCIENCE-DRIVEN THERAPIES TO TARGET HEALTHSPAN WITH AGING: CLINICAL IMPLICATIONS

**DOI:** 10.1093/geroni/igae098.1910

**Published:** 2024-12-31

**Authors:** Sara Espinoza, Mitzi Gonzales, Nicolas Musi

**Affiliations:** Cedars-Sinai Medical Center, Los Angeles, California, United States; Cedars-Sinai Medical Center, Los Angeles, California, United States; Cedars-Sinai Medical Center, Los Angeles, California, United States

## Abstract

The geroscience hypothesis posits that by targeting aging we may simultaneously prevent or delay several age-related diseases and thereby increase healthspan, or lifespan spent free of significant disease and disability. Clinical trials of candidate agents are underway to examine several pharmacological interventions for this purpose. Beyond determining the safety of these agents, substantial work will be needed to determine the efficacy of these interventions on various domains of healthspan, i.e., physical function, cognitive function, frailty, disability, patient reported outcomes and health-related quality of life. Further, we will need consensus to identify the most meaningful and clinically relevant outcomes and biomarkers of target engagement to identify appropriate patient populations and monitor therapeutic response. It is likely that no single intervention will be broadly indicated, but agents will be tailored using an individualized, patient-centered approach. Larger pragmatic clinical trials will be needed to demonstrate efficacy beyond smaller, early phase clinical trials. Furthermore, any interventions for older adults must not inadvertently exacerbate existing medical comorbidities or geriatric syndromes, such as contribute to polypharmacy or cause side effects that outweigh any potential benefits. This will require that physicians and others providing medical care to older adults, the vast majority of which are not geriatricians, must be trained and knowledgeable in basic principles of geroscience and the use of such therapies toward the goal of improving healthspan (as opposed to the traditional single disease approach). Clinical practice guidelines specific to promoting healthspan in older adults may be needed to guide any future broad clinical use.

